# Radioimmunotherapy as a targeted strategy of reduced-intensity conditioning for allogeneic transplantation in leukemia

**DOI:** 10.3389/fonc.2026.1846420

**Published:** 2026-06-23

**Authors:** Phuong T. Vo, Johnnie J. Orozco, Brenda M. Sandmaier

**Affiliations:** 1 Clinical Research Division, Fred Hutch Cancer Center, Seattle, WA, United States; 2 Division of Hematology and Oncology, Department of Medicine, University of Washington School of Medicine, Seattle, WA, United States; 3 Translational Science & Therapeutics Division, Fred Hutch Cancer Center, Seattle, WA, United States

**Keywords:** allogenic, conditioning, hematopoietic stem cell transplantation, leukemia, radioimmunotherapy

## Abstract

Reduced-intensity conditioning (RIC) has expanded the use of allogeneic hematopoietic transplant (allo-HCT) as a potentially curative treatment of a variety of hematological cancers including acute myeloid leukemia (AML) by reducing treatment-related morbidity and mortality. However, the RIC allo HCT success is limited by relapse, especially for patients with adverse molecular/cytogenetics or with measurable residual disease (MRD) at the time of HCT. Radioimmunotherapy (RIT) potentially improves the efficacy of RIC by enhancing the cytoreductive effect with the ability to deliver targeted radiation to sites of hematopoietic malignancy while sparing nonhematopoietic organs. In clinical trials using different combinations of targeting antibodies and radionuclides, reliable radiation targeting to marrow and spleen has been consistently demonstrated. Toxicity is often predictable and organ-specific with hepatic toxicity most commonly limiting the therapeutic dose. In this article, we review the rationale for combining RIT with RIC and discuss antigen selection and distinct properties of β- and α-emitting radionuclides. We also summarize clinical experience combining fludarabine-based RIC regimens with RIT and provide emerging directions, including the development of α-emitter platforms. Available data suggest that RIT may potentially improve disease control while maintaining the tolerability of RIC. Because the majority of the current evidence involves early-phase studies, additional prospective evaluation is needed before radioimmunotherapy can be considered a standard component of conditioning.

## 1 Introduction

Allogeneic hematopoietic cell transplantation (allo-HCT) remains the most effective treatment for patients with AML and other myeloid neoplasms (1, 2). Reduced-intensity conditioning (RIC) has extended the use of allo-HCT to older patients and those with significant comorbidities (3, 4). However, relapse remains the main limitation, especially in patients with adverse cytogenetics, high-risk molecular findings, or measurable residual disease (MRD) at transplantation (5–7).

A major challenge in RIC allo-HCT is how to increase antileukemic effects without forfeiting the regimens’ tolerability (2–4). RIT offers a unique approach by conjugating monoclonal antibodies to therapeutic radionuclides, delivering radiation specifically to hematopoietic tissues and sites of leukemia while minimizing exposure to nonhematopoietic organs (8–16).

CD45 is the most widely studied and used RIT target. The surface marker CD45 is broadly expressed on hematopoietic cells including 85%-90% of AML cells with minimal expression on nonhematopoietic tissues. This selective expression pattern permits the monoclonal antibody to deliver radiation specifically to marrow and lymphoid compartments (8–10, 12, 14, 17, 18). Other targets, including CD33 and CD66, have also been studied, although with more restricted applicability (13, 18–22). Clinical studies investigating RIT in combination with fludarabine-based RIC regimens have demonstrated feasibility, and consistent ability to target radiation to hematopoietic tissues (10, 12–14, 18, 22, 23). Hepatic complications such as veno-occlusive disease/sinusoidal obstruction syndrome (VOD/SOS) have been the main therapeutic dose limitation, highlighting the importance of organ-specific dose assessment (10, 12, 14, 16, 18).

Recently researchers have shifted their interest from β-emitters such as iodine-131 and yttrium-90 to α-emitters such as astatine-211, which provide more localized, high–linear-energy-transfer (LET) radiation and may be particularly relevant in low-burden or MRD-positive disease (24–33). By further eliminating leukemic burden before donor engraftment, RIT-based conditioning may potentially facilitate more effective graft-versus-leukemia (GVL) without increasing the risk of graft-versus-host disease.

In this brief review, we summarize the rationale for combining RIT into RIC allo-HCT for leukemia, including main principles of target antigen selection, radiation delivery, and radionuclide choice (15, 16, 18). We then review the clinical experience with particular attention to feasibility, potential efficacy, organ-specific toxicity, and emerging strategies such as the use α-emitters (18, 22, 23, 34). Despite success with RIT before allo-HCT, the role of RIT-based conditioning must be considered within the evolving therapeutic landscape of AML and related myeloid malignancies to further improve the impact of RIT. Increasingly, MRD is used to guide transplant decision-making, including timing of transplantation and selection of conditioning intensity. In parallel, the incorporation of targeted therapies such as FLT3 and IDH inhibitors, as well as venetoclax-based regimens, has improved disease control prior to allo-HCT. Post-transplant strategies, including maintenance therapy and cellular immunotherapies, are also being explored to reduce relapse risk. In this context, the role of RIT is as an approach for targeted cytoreduction, particularly in patients with residual disease at the time of transplantation.

## 2 Biological rationale for RIT-augmented reduced-intensity conditioning

RIC regimens rely predominantly on GVL effects to compensate for reduced cytoreductive intensity (1, 3). RIT offers a very distinctive strategy to intensify RIC by delivering targeted radiation to hematopoietic and leukemia cells, enhancing cytoreduction while preserving the tolerability of RIC in addition to other potential biologic effects (15, 16, 18).

### 2.1 Targeted radiation versus conventional total body irradiation

#### 2.1.1 Dose localization

Conventional total body irradiation (TBI) used for allo-HCT conditioning delivers similar radiation exposure to malignant tissues and normal organs. The use of high-dose TBI increases the treatment-related mortality because it severely damages multiple organs such as lungs, liver, kidneys, and gastrointestinal tract (16). Historical randomized studies from Seattle showed that AML patients who received escalating TBI doses to approximately 15–16 Gy had lower relapse rates but higher treatment-related mortality, subsequently leading to no overall survival benefit compared with those who received standard-dose TBI (35–37). In contrast, RIT allows radiation to be targeted to specific antigens, making RIT more specific to the bone marrow, spleen and lymphoid organs, as demonstrated by studies looking at the distribution of CD45- and CD66- RIT (8–10, 12–14, 16).

#### 2.1.2 Microdosimetry and spatial heterogeneity

At the cellular level, RIT has microdosimetric advantages over TBI. Radioactivity delivered by antibody-bound radionuclides is extremely localized, but this should be balanced by taking into account antigen expression, antibody binding, and the path length of emitted particles (16, 29). The phenomenon of cross-fire may also expose other potentially radiosensitive cells in the marrow niche, so that the radiation may be aimed at leukemic or lymphoma-infiltrated marrow while injuring normal cells in the niche (29, 30). Such advantages are hard to achieve with external-beam radiation, demonstrating the unique potential of RIT-based conditioning (16, 18). One potential advantage of radioimmunotherapy-based conditioning is that it may enhance GVL effects indirectly by reducing residual disease burden before donor cell infusion and by modifying the marrow microenvironment in ways that favor donor immune surveillance (38, 39). By decreasing leukemic burden in the marrow and spleen, targeted radiation may reduce the pool of malignant cells capable of escaping early post-transplant immune control. Radiation-induced changes in the niche may also influence antigen presentation, cellular trafficking, and local inflammatory signaling, thereby increasing the susceptibility of residual disease to graft-mediated immune eradication (38, 39). In addition, depletion of host hematopoietic and immune compartments may reduce host-versus-graft resistance and facilitate donor immune engraftment (24, 25, 40–45).

### 2.2 RIT target antigens in leukemia

#### 2.2.1 CD45 target

Although many antigens have been identified as possible targets for RIT of patients with hematological malignancies, CD45 is one of the most-studied antigens for RIT-based conditioning. It is found only on nucleated hematopoietic cells, including 85%-90% of AML cells and is mostly absent from nonhematopoietic tissues (17). With this expression pattern, radiation can be directed to marrow and lymphoid compartments (8–10, 12, 14). CD45’s high-density allows sustained radionuclide retention and efficient cross-fire within marrow spaces. In contrast, lower-density or rapidly internalizing antigens may limit the delivered dose (16, 29). Importantly, CD45 expression is still mostly preserved in relapsed and refractory disease that has been heavily treated prior to HCT (17). From a conditioning perspective, CD45-directed RIT can be helpful by both contributing to host lympho-myelosuppression and delivering antileukemic radiation (1, 18).

#### 2.2.2 CD33, CD66, and others

Other AML surface antigens have been studied but with limited applicability. CD33 is specific to myeloid cells but has variable expression and could be downregulated after antibody-targeted induction treatments (20–22). Early clinical studies also investigated CD33-targeted RIT using iodine-131–labeled anti-CD33 antibodies (M195). The study demonstrated the feasibility of this approach and showed antileukemic activity, including in patients with acute promyelocytic leukemia treated in remission (46). However, unstable antigen expression and the more frequent use of other CD33-targeted pre-HCT therapies have limited its development as part of allo-HCT conditioning. CD66 antigens have also been successfully used in European RIT-HCT studies (13). While these approaches provide important proof-of-concept, none have similar consistency, and broad coverage of CD45 for conditioning-based RIT (17, 18).

### 2.3 Radionuclide selection

#### 2.3.1 β-emitters: iodine-131 and yttrium-90

β-emitting radionuclides have been utilized most extensively in RIT clinical trials for allo-HCT. Iodine-131 and yttrium-90 emit electrons with long path lengths (measured in millimeters), which facilitate cross-fire irradiation and promote relatively uniform dose delivery within marrow spaces (18, 29). These characteristics can be beneficial in cases of bulky disease or when antigen expression is heterogeneous, but may be less effective in the setting of minimal residual disease or small clusters of circulating cells (16, 29). The longer path length of β-particles may also potentially increase the risk of off-target tissue injury (10, 12, 14, 16).

#### 2.3.2 α-emitters: astatine-211

Astatine-211 delivers LET radiation over very short distances (measured in micrometers), leading to significant and irreparable DNA damage with minimal cross-fire to surrounding tissues. Alpha particles deposit substantially more energy per decay (approximately 6–8 MeV) than beta emitters such as iodine-131 (about 0.6 MeV), resulting in greater local energy deposition and a higher likelihood of lethal DNA double-strand breaks (29–32). These features make α-emitters particularly suitable for settings with low disease burden or MRD, where precise targeting is essential (24–28, 33). The short path length also limits radiation exposure to nearby normal cells, potentially reducing off-target effects compared with β-emitters. This suggests that α-emitters may present a lower risk of long-term radiation-related secondary malignancies, although this remains theoretical given the limited long-term clinical data. The high potency of α-particles results in a relatively narrow therapeutic window, requiring careful activity selection and close monitoring for toxicity (25, 28, 30, 31).

**Figure 1:**
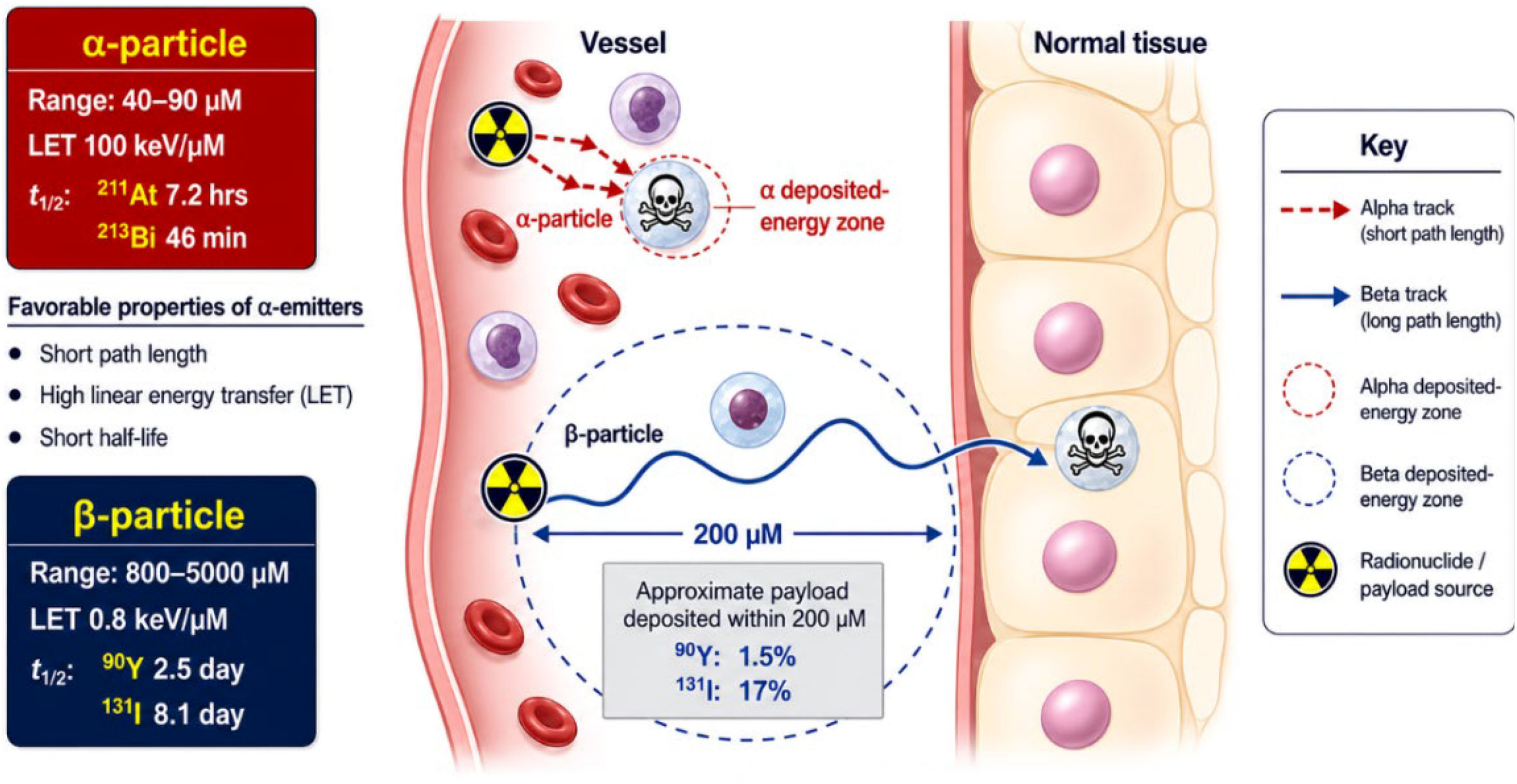
Schematic comparison of β- and α-emitting radionuclides used in radioimmunotherapy-based conditioning.

The key radiobiologic properties of radionuclides are summarized in **Table 1** and illustrated schematically in **Figure 1**.

**Table 1:** Physical and radiobiologic properties of radionuclides used in RIT.

Radionuclide	Emission type	Physical half-life	Representative particle energy	Approximate path length in tissue	Selected clinical implications
Iodine-131	β (γ co-emission)	~8 days	~0.6 MeV (mean β energy)	up to several millimeters	Enables cross-fire irradiation and imaging-based activity estimation; requires radiation-safety isolation procedures
Yttrium-90	β	~64 hours	up to ~2.3 MeV (maximum β energy)	several millimeters	Higher β energy with greater cross-fire range; lack of γ emission limits direct imaging
Astatine-211	α	~7.2 hours	~6–8 MeV (α particle energy per decay)	~50–80 µm	Very high LET and highly localized energy deposition; suited for low-burden disease targeting; requires rapid radiochemistry and coordinated clinical delivery
Bismuth-213	1α, 2β	46 min	5.84 (α)	~85 µm (from α daughter)	High LET but limited by very short half life
Actinium-225	4α, 2β	9.9 days	5.8-8.4 MeV	~40–100 µm	High LET but with long half life may contribute to prolonged myelosuppression in the marrow

## 3 Clinical experience with radioimmunotherapy combined with reduced-intensity conditioning

### 3.1 CD45-directed RIT combined with RIC

In one of Pagel et al.’s earliest clinical trials, 58 patients (median age 63 years) with advanced stage AML or high-risk MDS were treated with iodine-131 -anti-CD45 antibody (BC8) followed by fludarabine and 2 Gy TBI as conditioning prior to allo-HCT (10). These patients had active AML beyond 1st remission or MDS with excess blasts. The average maximum tolerated dose to the liver was calculated as approximately 24 Gy. All patients engrafted with rapid donor chimerism. Estimated 1-year relapse rate was 40% despite high-risk disease and estimated overall survival rate was 41% (10). These relapse rates, although observed in high-risk populations with refractory disease, underscore that RIT-based intensification may not fully overcome post-transplant disease recurrence.

Mawad et al. subsequently used the same approach in a younger cohort of 15 patients with advanced AML and high-risk MDS using iodine-131 -anti-CD45 and fludarabine with 2Gy TBI (12). Patients received between 332 and 1561 mCi, leading to average absorbed doses of 27 Gy to marrow, 84 Gy to spleen, and 21 Gy to liver. Some liver doses reached 28 Gy, but no dose-limiting toxicity was seen. Transplant outcomes were also favorable, with a 73% 1-year overall survival: 8 out of 15 patients had disease relapse (at a median of 54 days), and 40% were alive at a median of 5 years, with 0% NRM during the first year after transplant, supporting the tolerability and potential efficacy of RIT-based RIC (12).

CD45-targeted β-emitters have recently been studied with yttrium-90-DOTA-BC8, yttrium-90-conjugated anti-CD45 antibody. A phase I trial by Vo et al., treated 15 patients with relapsed or refractory leukemia or high-risk MDS with yttrium-90-DOTA-BC8 followed by fludarabine/2 Gy TBI RIC allo-HCT (14). 10 had advanced AML, and 5 had high-risk MDS, with 9 having active marrow disease and blast counts from 7% to 84%. As no dose-limiting toxicity was observed, the maximum tolerated dose was never reached (up to 30 Gy to the liver). All patients engrafted by 28 days. 13/15 (87%) patients achieved CR at 28 days. At a median follow-up of 1.8 years, the overall survival was 66% and 46% at 1 and 2 years, with the probability of progression-free survival of 46% at 1 and 2 years, respectively (14).

One previous phase I/II study also incorporated CD45-directed RIT into a myeloablative conditioning regimen. Pagel et al. combined iodine-131-labeled anti-CD45 antibody with busulfan and cyclophosphamide in patients with AML in first remission, demonstrating acceptable regimen-related toxicity despite the addition of a large dose of hematopoietic radiation from RIT (9). Taken together, these studies indicate that RIT did not increase treatment related toxicity at this dose and may be safely combined with reduced-intensity and myeloablative conditioning. The phase III SIERRA trial compared iodine-131-apamistamab (Iomab-B) with standard therapy in 55 years or older patients with active relapsed or refractory AML ineligible for standard myeloablative conditioning. Patients were randomized to receive Iomab-B and subsequently allo-HCT, or standard therapy, with the option to crossover after completion of standard therapy. In the Iomab-B arm, 22% of patients achieved a 6-month durable complete remission compared to 0% in the control arm. Non-relapse mortality in the first 100 days post-transplant was considered low (6%), and the data suggest that CD45-directed RIT is a safe transplant conditioning approach in patients with active AML, with targeted radiation improving allo-HCT accessibility and disease control, without substantial toxicities (47, 48).

In summary, iodine-131-BC8 and yttrium-90-DOTA-BC8 provide the best clinical evidence for CD45-directed β-emitter RIT with RIC allo-HCT, and the non-relapse mortality was comparable to other RIC cohorts, indicating that combining RIT with additional targeted radiation to RIC maintains the regimen tolerability (9, 10, 12–14, 16).

### 3.2 CD66-directed RIT combined with RIC

Koenecke et al. reported the results of allo-HCT in 21 patients with high-risk myeloid malignancies (AML and MDS) who were conditioned with Rhenium-188 -labeled anti-CD66 antibodies before transplant (13). Eleven patients were treated with myeloablative conditioning and 10 with reduced-intensity conditioning. CD66-directed RIT could be utilized with both myeloablative and reduced-intensity conditioning regimens. With a median follow up of 42 months (23–60), disease free survival was 43% across the entire study. Treatment related mortality was 28.6%. Later series from Europe evaluated this approach in the properly reduced-intensity transplant setting. In a prospective phase II series, 22 elderly patients (median age 65 years) with advanced myeloid malignancy received anti-CD66 radioimmunotherapy followed by fludarabine, busulfan, and alemtuzumab (fludarabine-Bu-alemtuzumab) as conditioning for allo-HCT (13, 18). This regimen was feasible, with good engraftment but high rates of relapse (40%). Though CD66-targeted RIT has demonstrated marrow-targeted radiation which may be combined with several conditioning regimens in HCT, the fact that its target is only present in certain subset of cell types and varies in expression between leukemia types has made it less investigated than the more ubiquitous CD45.

Although these studies provide consistent evidence that radioimmunotherapy can be integrated into reduced-intensity conditioning with reliable engraftment and acceptable acute toxicity, the available clinical data remains limited in scope. Most reports are early-phase, single-center studies with small sample sizes and heterogeneous patient populations, including variation in age, disease status, prior therapy, and conditioning backbone. Additional larger multicenter studies with appropriate comparison groups will be required to clarify the clinical value of this approach.

### 3.3 Toxicity patterns and dose-limiting organs

Prior studies used dosimetry to determine the biodistribution of antibody and the radiation absorbed dose to specific organs. RIT dosimetry typically used a tracer or imaging dose of radiolabeled antibody given prior to conditioning. Serial whole-body and organ-specific imaging, combined with blood clearance measurements, allowed estimation of radiation absorbed dose to marrow and critical organs (10, 12, 14).

The liver has been the dose-limiting organ in all anti-CD45 RIT studies, probably because of hepatic radiation exposure from a combination of uptake of radioimmunoconjugate by both the Kupffer cells and other resident hematopoietic-derived cells of the liver, nonspecific antibody clearance of radioimmunoconjugate from the circulation, and catabolism of radiolabeled antibody complexes (16). In the original iodine-131-BC8 dose escalation trial , combined with fludarabine and 2 Gy, the MTD for liver was estimated at 24 Gy (10). Subsequent studies in younger or fit patients have tolerated 28–30 Gy without any definitive dose-limiting liver toxicity, though hepatic injury, including transaminitis and hyperbilirubinemia, as well as VOD/SOS remain the most clinically relevant toxicities (12, 14). Hepatic toxicity can also be late in onset, and therefore not recognized directly within classic dose-limiting hematologic and gastrointestinal toxicity time windows, but rather occurring several weeks to months after transplantation. This may indicate the need for longer follow-up in RIT dose-escalation studies to ensure safety (16). These findings are clinically relevant in RIC HCT, where most patients may be older, and/or have a high burden of comorbidities and prior toxic exposures, including cytotoxic chemotherapy, transfusion-related iron overload and/or liver disease (4).

In contrast to hepatic exposure, the absorbed doses to the kidneys and lungs involved in leukemia-targeted RIT have historically been lower than the liver due to relatively low retention of antibodies in these tissues (10, 12, 14), and there have been no reports of clinically meaningful renal or pulmonary toxicity in CD45-targeted RIT clinical trials.

Some studies suggested that significant hepatic radiation exposure could be driven in part by circulating blood-pool activity, rather than fixed tissue binding alone. This observation has provided a mechanistic rationale for approaches such as pretargeted RIT, in which a non-radioactive antibody is given first and allowed to clear, followed by a small radioactive ligand to minimize off-target radiation exposure (16, 26).

Importantly, hematopoietic recovery has remained protected despite this high marrow absorbed dose. In the iodine-131-BC8 (12) and yttrium-90-DOTA-BC8 (14) data subsets, engraftment has been achieved in all patients, with graft failure not observed even in the setting of high marrow absorbed doses of 25–40 Gy or greater (12, 14).

Beyond hepatic toxicity, other clinically relevant adverse events should be considered, including graft-versus-host disease, infectious complications, and late organ toxicities. There is no evidence that radioimmunotherapy increases the incidence or severity of graft-versus-host disease. Similarly, while infection risk is a major concern in this population, studies to date do not show different infectious safety signals compared to standard conditioning approaches. In addition, long-term and late toxicities, including secondary malignancies and chronic organ dysfunction, remain incompletely characterized. Given the targeted and non-uniform radiation distribution associated with RIT, the long-term risk profile may differ from conventional conditioning approaches and requires further study with extended follow-up.

Overall, for most RIT approaches, the dose-limiting toxicity is not the ablation of the marrow, but off-target toxicity to the liver. While these studies collectively demonstrate the feasibility of incorporating RIT into RIC platforms, most available data derive from early-phase, single-center studies with small sample sizes and heterogeneous patient populations, including variability in disease status at transplantation, prior therapies, and conditioning backbones. These factors introduce potential selection bias and limit cross-study comparability. In addition, many cohorts have been treated at specialized centers with expertise in dosimetry and radiopharmaceutical delivery, which may impact generalizability. The randomized phase 3 SIERRA trial demonstrated that iodine-131-apamistamab–based conditioning can improve outcomes compared with conventional care in older adults with relapsed/refractory AML, providing important proof-of-concept for RIT-enabled transplantation (47). However, additional prospective studies are still needed to define the role of RIT across conditioning platforms, disease states, radionuclides, and post-transplant therapeutic strategies.

## 4 Emerging strategies

### 4.1 Alpha-emitter RIT

More recently, the α-emitting radionuclide astatine-211 has been investigated as a potential next generation CD45 RIT isotope because of its short path length and high LET that improve localized cytotoxicity while minimizing cross-fire, potentially useful in the low-burden or MRD-positive setting (22, 29–32).

Preclinical studies provide the primary biologic rationale for α-emitter RIT. In murine models of disseminated AML, astatine-211-labeled anti-CD45 antibodies targeted hematopoietic radiation facilitated donor marrow engraftment and prolonged survival following transplantation (24). Early studies comparing the relative myelosuppression and toxicities in mice treated with anti-CD45 antibody labeled with the alpha emitters astatine-211 or bismuth-213 showed that lower activities of astatine-211 on anti-CD45 antibody were adequately myelosuppressive and myeloablative with less non-hematologic toxicity than bismuth-213 (25). Preclinical studies indicated that conditioning with astatine-211 labeled anti CD45 antibodies provided sufficient lympho- and myelosuppression for engraftment (24, 25, 41–45).

Clinical experience with alpha-emitters for AML. Despite these encouraging preclinical findings, clinical experience with alpha-emitter radioimmunotherapy in the transplant setting remains very limited. Clinical experience with α-particle RIT outside of the transplant setting has demonstrated potential antileukemic effects. In a phase I/II trial of cytarabine plus α-emitter-labeled anti-CD33 antibody bismuth-213-lintuzumab, 19% overall response rates were seen at the maximum tolerated dose (37 MBq/kg) in relapsed AML patients (20). In a first-in-human trial of actinium-225 -lintuzumab, acceptable safety and reduction of bone marrow blast counts was observed in >65% of patients with relapsed/refractory disease (49). In a separate trial of actinium-225 -lintuzumab in older patients with previously untreated AML, evidence of substantial antileukemic activity of the agent was observed, with response rates as high as 2/3 of treated patients (50). However, higher doses have been associated with cytopenias persisting for prolonged periods, delayed marrow recovery, and transfusion dependence owing to prolonged half-life and therefore sometimes requiring dose reduction (20). These results show the challenge of balancing the potency versus the hematologic toxicity of high LET radiation and suggest that the use of targeted radioimmunotherapy is best done in the setting of allogeneic transplant. Additionally, the use of an α-emitter with a shorter half-life, such as astatine-211, may be suitable for allo-HCT conditioning.

Early experience with astatine-211 in allo-HCT gave preliminary evidence for the feasibility of this approach. Sandmaier et al. presented at the annual American Society for Transplantation and Cellular Therapy (ASTCT) meeting in 2021: 20 patients with AML and high risk MDS (most of them in active disease at the time of allo-HCT) received astatine-211 labeled anti-CD45 antibody followed by fludarabine and low-dose TBI. This report shows high engraftment rates and promising outcomes, with 1- and 2-year disease-free survival of 43% and 38%, and overall survival of 47% and 42% respectively (28). The results suggest that α-emitter RIT may improve disease control without compromising tolerability of the RIC backbone, but further clinical data are needed.

Taken together, clinical experience with α-emitter–based RIT in the allo-HCT conditioning setting remains limited to small, early-phase studies, and much of the supporting rationale derives from preclinical models. Translation into broader clinical practice is further constrained by logistical challenges, including radionuclide production and the need for highly coordinated radiochemistry and delivery infrastructure. In addition, the high linear energy transfer of α-particles, while potentially advantageous for tumor cell killing, requires careful dose optimization to balance antileukemic activity, host lympho-myelosuppression, donor engraftment, and nonhematologic toxicity. Further prospective studies are needed to define the optimal dose, safety profile, and clinical benefit of α-emitter RIT as part of allo-HCT conditioning.

Key prospective and translational clinical studies RIT-based conditioning regimens are summarized in **Table 2**.

**Table 2:** Selected clinical studies of RIT conditioning.

Study	Target antigen	Radionuclide	Conditioning backbone	Key radiation-distribution/safety observations	Clinical outcomes
Matthews et al., 1999 (8)	CD45	iodine-131	Biodistribution/dosimetry study	Preferential uptake in marrow and spleen with favorable marrow-to-organ dose ratios	Established feasibility of hematopoietic-targeted radiation delivery
Pagel et al., 2009 (10)	CD45	iodine-131	Fludarabine + 2 Gy TBI + CD45 RIT	Liver dose defined therapeutic window; maximum tolerated dose ≈ 24 Gy to liver in elderly patients	1-year relapse ≈ 40%; 1-year overall survival 41%
Mawad et al., 2014 (12)	CD45	iodine-131	Fludarabine + 2 Gy TBI + CD45 RIT	Mean absorbed doses: 27 Gy marrow, 84 Gy spleen, 21 Gy liver; liver doses up to 28 Gy tolerated in younger population	1-year OS 73%; relapse in 8/15 patients; no 1-year NRM
Koenecke et al., 2008 (13)	CD66	¹^88^Re	CD66-RIT + allo-HCT (mixed conditioning)	Effective marrow localization with acceptable organ exposure	Demonstrated feasibility; relapse remained frequent
European CD66 RIT studies (18)	CD66	β-emitter platforms	Fludarabine (150 mg/m(2)), busulfan (8 mg/kg) and alemtuzumab (75 mg)	Demonstrated marrow-directed radiation delivery in elderly patients	Relapse ≈ 40%
Vo et al., 2020 (14)	CD45	yttrium-90	Fludarabine + 2 Gy TBI + CD45 RIT	Liver doses reached ≈30 Gy without identifying maximum tolerated dose	Complete remission 87%; 1-year OS 66%; 2-year OS 46%
Sandmaier et al., 2021 (28)	CD45	astatine-211	Fludarabine + TBI + α-RIT	First-in-human α-emitter conditioning	Early clinical feasibility reported
SIERRA Trial 2025 (47)	CD45	iodine-131	Fludarabine + 2 Gy TBI + CD45 RIT	Targeted radiation enables transplantation in active AML	Improved transplant access and remission in relapsed/refractory AML with some long-term responders

### 4.2 Novel targets and antibody engineering

Other improvements to RIT-based conditioning may occur through improving antibody affinity, antibody or Fc engineering, or identification of new antigens beyond CD45 to widen the range of targetable antigens for RIT-based conditioning (17, 33, 51–55). Potential preclinical targets for leukemia immunotherapy that have been evaluated in preclinical studies include CD123 (the interleukin-3 receptor α chain, expressed on leukemic stem cells in some patients with AML) for which the first α-particle RIT studies have been performed in xenografts (33), and CXCR4 which has been evaluated in terms of preclinical pharmacokinetics and dosimetry in leukemia (51).

However, reproducible expression across disease states is a requirement for any new target to be a viable replacement or addition to CD45-based platforms for effective conditioning regimens, as consistent localization of the target to the marrow is critical (17, 18, 22, 23).

## 5 Conclusion

Reduced-intensity conditioning has extended HCT access to many more patients with leukemia but relapse remains the main cause of treatment failure (1, 4, 6). Radioimmunotherapy offers a unique approach to enhance antileukemic cytoreduction through targeted radiation delivery while maintaining the tolerability of RIC (18, 22, 23). Clinical studies to date show that this strategy is feasible, can support reliable engraftment, and produces organ-specific toxicity profiles in which hepatic exposure is the principal limitation. However, the current evidence base is derived primarily from phase I/II trials and definitive comparisons with standard conditioning approaches are still lacking. Additional prospective multicenter studies and longer follow-up will be required to define the optimal role of radioimmunotherapy in transplant conditioning and to determine whether its biologic promise translates into durable clinical benefit.

## References

[1] Appelbaum FR . Haematopoietic cell transplantation as immunotherapy. Nature. (2001) 411:385–9. doi: 10.1038/35077251 11357147

[2] Gyurkocza B Sandmaier BM . Conditioning regimens for hematopoietic cell transplantation: one size does not fit all. Blood. (2014) 124:344–53. doi: 10.1182/blood-2014-02-514778 24914142 PMC4102707

[3] McSweeney PA Niederwieser D Shizuru JA Sandmaier BM Molina AJ Maloney DG . Hematopoietic cell transplantation in older patients with hematologic Malignancies: replacing high-dose cytotoxic therapy with graft-versus-tumor effects. Blood. (2001) 97:3390–400. doi: 10.1182/blood.v97.11.3390 11369628

[4] Sorror ML Maris MB Storb R Baron F Sandmaier BM Maloney DG . Hematopoietic cell transplantation (HCT)-specific comorbidity index: a new tool for risk assessment before allogeneic HCT. Blood. (2005) 106:2912–9. doi: 10.1182/blood-2005-05-2004 15994282 PMC1895304

[5] Walter RB Buckley SA Pagel JM Wood BL Storer BE Sandmaier BM . Significance of minimal residual disease before myeloablative allogeneic hematopoietic cell transplantation for AML in first and second complete remission. Blood. (2013) 122:1813–21. doi: 10.1182/blood-2013-06-506725 23847197 PMC3765060

[6] Araki D Wood BL Othus M Radich JP Halpern AB Zhou Y . Allogeneic hematopoietic cell transplantation for acute myeloid leukemia: time to move toward a minimal residual disease-based definition of complete remission? J Clin Oncol. (2016) 34:329–36. doi: 10.1182/blood.v126.23.2571.2571 PMC4872033 26668349

[7] Walter RB Potter V Craddock C . Allogeneic hematopoietic cell transplantation for acute myeloid leukemia in adults over 70 years old. Blood. (2025) 145:2847–56. doi: 10.1182/blood.2024024247 39241195 PMC12226756

[8] Matthews DC Appelbaum FR Eary JF Fisher DR Durack LD Hui TE . Phase I study of (131)I-anti-CD45 antibody plus cyclophosphamide and total body irradiation for advanced acute leukemia and myelodysplastic syndrome. Blood. (1999) 94:1237–47. doi: 10.1182/blood.v94.4.1237 10438711

[9] Pagel JM Appelbaum FR Eary JF Rajendran J Fisher DR Gooley T . 131I-anti-CD45 antibody plus busulfan and cyclophosphamide before allogeneic hematopoietic cell transplantation for treatment of acute myeloid leukemia in first remission. Blood. (2006) 107:2184–91. doi: 10.1182/blood-2005-06-2317 16254140 PMC1895719

[10] Pagel JM Gooley TA Rajendran J Fisher DR Wilson WA Sandmaier BM . Allogeneic hematopoietic cell transplantation after conditioning with 131I-anti-CD45 antibody plus fludarabine and low-dose total body irradiation for elderly patients with advanced acute myeloid leukemia or high-risk myelodysplastic syndrome. Blood. (2009) 114:5444–53. doi: 10.1182/blood-2009-03-213298 19786617 PMC2798861

[11] Press OW Howell-Clark J Anderson S Bernstein I . Retention of B-cell-specific monoclonal antibodies by human lymphoma cells. Blood. (1994) 83:1390–7. doi: 10.1182/blood.v83.5.1390.1390 8118040

[12] Mawad R Gooley TA Rajendran JG Fisher DR Gopal AK Shields AT . Radiolabeled anti-CD45 antibody with reduced-intensity conditioning and allogeneic transplantation for younger patients with advanced acute myeloid leukemia or myelodysplastic syndrome. Biol Blood Marrow Transplant. (2014) 20:1363–8. doi: 10.1016/j.bbmt.2014.05.014 24858425 PMC4127337

[13] Koenecke C Hofmann M Bolte O Gielow P Dammann E Stadler M . Radioimmunotherapy with [188Re]-labelled anti-CD66 antibody in the conditioning for allogeneic stem cell transplantation for high-risk acute myeloid leukemia. Int J Hematol. (2008) 87:414–21. doi: 10.1007/s12185-008-0043-1 18415659

[14] Vo P Gooley TA Rajendran JG Fisher DR Orozco JJ Green DJ . Yttrium-90-labeled anti-CD45 antibody followed by a reduced-intensity hematopoietic cell transplantation for patients with relapsed/refractory leukemia or myelodysplasia. Haematologica. (2020) 105:1731–7. doi: 10.3324/haematol.2019.229492 31582553 PMC7271581

[15] Larson SM Carrasquillo JA Cheung NK Press OW . Radioimmunotherapy of human tumours. Nat Rev Cancer. (2015) 15:347–60. doi: 10.1038/nrc3925 25998714 PMC4798425

[16] Pouget JP Navarro-Teulon I Bardies M Chouin N Cartron G Pelegrin A . Clinical radioimmunotherapy--the role of radiobiology. Nat Rev Clin Oncol. (2011) 8:720–34. doi: 10.1038/nrclinonc.2011.160 22064461

[17] Dahlke MH Larsen SR Rasko JE Schlitt HJ . The biology of CD45 and its use as a therapeutic target. Leuk Lymphoma. (2004) 45:229–36. doi: 10.1080/1042819031000151932 15101706

[18] Lauter A Strumpf A Platzbecker U Schetelig J Wermke M Radke J . 188Re anti-CD66 radioimmunotherapy combined with reduced-intensity conditioning and in-vivo T cell depletion in elderly patients undergoing allogeneic haematopoietic cell transplantation. Br J Haematol. (2010) 148:910–7. doi: 10.1111/j.1365-2141.2009.08025.x 19995390

[19] Bodet-Milin C Kraeber-Bodere F Eugene T Guerard F Gaschet J Bailly C . Radioimmunotherapy for treatment of acute leukemia. Semin Nucl Med. (2016) 46:135–46. doi: 10.1053/j.semnuclmed.2015.10.007 26897718

[20] Rosenblat TL McDevitt MR Mulford DA Pandit-Taskar N Divgi CR Panageas KS . Sequential cytarabine and alpha-particle immunotherapy with bismuth-213-lintuzumab (HuM195) for acute myeloid leukemia. Clin Cancer Res. (2010) 16:5303–11. doi: 10.14694/edbook_am.2014.34.e126 20858843 PMC2970691

[21] Nikula TK McDevitt MR Finn RD Wu C Kozak RW Garmestani K . Alpha-emitting bismuth cyclohexylbenzyl DTPA constructs of recombinant humanized anti-CD33 antibodies: pharmacokinetics, bioactivity, toxicity and chemistry. J Nucl Med. (1999) 40:166–76. 9935073

[22] Walter RB . Where do we stand with radioimmunotherapy for acute myeloid leukemia? Expert Opin Biol Ther. (2022) 22:555–61. doi: 10.1080/14712598.2022.2060735 35350938 PMC9090441

[23] Green DJ Press OW . Whither radioimmunotherapy: to be or not to be? Cancer Res. (2017) 77:2191–6. doi: 10.1158/0008-5472.can-16-2523 28428282 PMC5413412

[24] Orozco JJ Back T Kenoyer A Balkin ER Hamlin DK Wilbur DS . Anti-CD45 radioimmunotherapy using (211)At with bone marrow transplantation prolongs survival in a disseminated murine leukemia model. Blood. (2013) 121:3759–67. doi: 10.1182/blood-2012-11-467035 23471305 PMC3643772

[25] Nakamae H Wilbur DS Hamlin DK Thakar MS Santos EB Fisher DR . Biodistributions, myelosuppression, and toxicities in mice treated with an anti-CD45 antibody labeled with the alpha-emitting radionuclides bismuth-213 or astatine-211. Cancer Res. (2009) 69:2408–15. doi: 10.1158/0008-5472.can-08-4363 19244101 PMC2657815

[26] Pagel JM Kenoyer AL Back T Hamlin DK Wilbur DS Fisher DR . Anti-CD45 pretargeted radioimmunotherapy using bismuth-213: high rates of complete remission and long-term survival in a mouse myeloid leukemia xenograft model. Blood. (2011) 118:703–11. doi: 10.1182/blood-2011-04-347039 21613259 PMC3142907

[27] Kornblit B Chen Y Sandmaier BM . Conditioning with alpha-emitter based radioimmunotherapy in canine allogeneic hematopoietic cell transplantation. Chimerism. (2012) 3:40–2. doi: 10.4161/chim.20726 22772070 PMC3442811

[28] Sandmaier BM Wilbur DS Hamlin DK Vo P Wong R Baker K . First-in-human astatine-211–BC8-B10 combined with fludarabine and total body irradiation conditioning: a dose-escalation study. Transplant Cell Ther. (2021) 27:S54. doi: 10.1016/S2666-6367(21)00083-X

[29] Behr TM Behe M Stabin MG Wehrmann E Apostolidis C Molinet R . High-linear energy transfer (LET) alpha versus low-LET beta emitters in radioimmunotherapy of solid tumors: therapeutic efficacy and dose-limiting toxicity of 213Bi- versus 90Y-labeled CO17-1A Fab' fragments in a human colonic cancer model. Cancer Res. (1999) 59:2635–43. 10363986

[30] Aghevlian S Boyle AJ Reilly RM . Radioimmunotherapy of cancer with high linear energy transfer (LET) radiation delivered by radionuclides emitting alpha-particles or Auger electrons. Adv Drug Delivery Rev. (2017) 109:102–18. doi: 10.1016/j.addr.2015.12.003 26705852

[31] Eychenne R Cherel M Haddad F Guerard F Gestin JF . Overview of the most promising radionuclides for targeted alpha therapy: the "Hopeful Eight. Pharmaceutics. (2021) 13:906. doi: 10.3390/pharmaceutics13060906 34207408 PMC8234975

[32] Wilbur DS . Enigmatic astatine. Nat Chem. (2013) 5:246. doi: 10.1038/nchem.1580 23422568

[33] Laszlo GS Orozco JJ Kehret AR Lunn MC Huo J Hamlin DK . Development of [(211)At]astatine-based anti-CD123 radioimmunotherapy for acute leukemias and other CD123+ Malignancies. Leukemia. (2022) 36:1485–91. doi: 10.1038/s41375-022-01580-7 35474099 PMC9177726

[34] Vago L Gojo I . Immune escape and immunotherapy of acute myeloid leukemia. J Clin Invest. (2020) 130:1552–64. doi: 10.1172/jci129204 32235097 PMC7108895

[35] Clift RA Buckner CD Appelbaum FR Bryant E Bearman SI Petersen FB . Allogeneic marrow transplantation in patients with chronic myeloid leukemia in the chronic phase: a randomized trial of two irradiation regimens. Blood. (1991) 77:1660–5. doi: 10.1182/blood.v77.8.1660.bloodjournal7781660 2015394

[36] Clift RA Buckner CD Appelbaum FR Sullivan KM Storb R Thomas ED . Long-term follow-up of a randomized trial of two irradiation regimens for patients receiving allogeneic marrow transplants during first remission of acute myeloid leukemia. Blood. (1998) 92:1455–6. doi: 10.1182/blood.v92.4.1455.spll2_1455_1456 9694737

[37] Clift RA Radich J Appelbaum FR Martin P Flowers ME Deeg HJ . Long-term follow-up of a randomized study comparing cyclophosphamide and total body irradiation with busulfan and cyclophosphamide for patients receiving allogenic marrow transplants during chronic phase of chronic myeloid leukemia. Blood. (1999) 94:3960–2. doi: 10.1182/blood.v94.11.3960a.423a43g_3960_3962 10627126

[38] O'Neill AT Chakraverty R . Graft versus leukemia: current status and future perspectives. J Clin Oncol. (2021) 39:361–72. doi: 10.1200/JCO.20.01801 33434054

[39] Hagen MW Setiawan NJ Dexter S Woodruff KA Gaerlan FK Billings TM . The bone marrow niche and hematopoietic system are distinctly remodeled by CD45-targeted astatine-211 radioimmunotherapy. Blood Adv. (2026) 10:2452–69. doi: 10.1182/bloodadvances.2025017065 41671449 PMC13083732

[40] Orozco JJ Kenoyer A Balkin ER Gooley TA Hamlin DK Wilbur DS . Anti-CD45 radioimmunotherapy without TBI before transplantation facilitates persistent haploidentical donor engraftment. Blood. (2016) 127:352–9. doi: 10.1182/blood-2014-12-617019 26576864 PMC4722286

[41] Chen Y Kornblit B Hamlin DK Sale GE Santos EB Wilbur DS . Durable donor engraftment after radioimmunotherapy using alpha-emitter astatine-211-labeled anti-CD45 antibody for conditioning in allogeneic hematopoietic cell transplantation. Blood. (2012) 119:1130–8. doi: 10.1182/blood-2011-09-380436 22134165 PMC3277350

[42] Burtner CR Chandrasekaran D Santos EB Beard BC Adair JE Hamlin DK . (211)Astatine-conjugated monoclonal CD45 antibody-based nonmyeloablative conditioning for stem cell gene therapy. Hum Gene Ther. (2015) 26:399–406. doi: 10.1089/hum.2015.021 25919226 PMC4492596

[43] Frost SH Miller BW Back TA Santos EB Hamlin DK Knoblaugh SE . Alpha-imaging confirmed efficient targeting of CD45-positive cells after 211At-radioimmunotherapy for hematopoietic cell transplantation. J Nucl Med. (2015) 56:1766–73. doi: 10.2967/jnumed.115.162388 26338894 PMC4675464

[44] Nakaya A Qiu H Santos EB Hamlin DK Wilbur DS Storb R . Addition of astatine-211-labeled anti-CD45 antibody to TBI as conditioning for DLA-identical marrow transplantation: a novel strategy to overcome graft rejection in a canine presensitization model: "Radioimmunotherapy to overcome transfusion-induced sensitization. Transplant Cell Ther. (2021) 27:476 e1–7. doi: 10.1016/j.jtct.2021.02.018 33775618 PMC8217096

[45] Frost SHL Orozco JJ Back TA Miller BW Santos EB Kenoyer A . (211)At-labeled anti-CD45 antibody as a nonmyeloablative conditioning for canine DLA-haploidentical stem cell transplantation. J Nucl Med. (2024) 65:1443–9. doi: 10.2967/jnumed.124.267540 39025648 PMC11372266

[46] Jurcic JG Caron PC Nikula TK Papadopoulos EB Finn RD Gansow OA . Radiolabeled anti-CD33 monoclonal antibody M195 for myeloid leukemias. Cancer Res. (1995) 55:5908s–10s. 7493368

[47] Gyurkocza B Nath R Seropian S Choe H Litzow MR Abboud C . Randomized phase III SIERRA trial of (131)I-apamistamab before allogeneic hematopoietic cell transplantation versus conventional care for relapsed/refractory AML. J Clin Oncol. (2025) 43:201–13. doi: 10.1200/jco.23.02018 39298738 PMC11709001

[48] Foran J Gyurkocza B Nath R Choe H Litzow MR Abboud C . 131I-apamistamab-led allogeneic hematopoietic cell transplant for patients with TP53 mutated R/R AML results in significantly improved outcomes. Transplant Cell Ther. (2024) 30:S110–1. doi: 10.1016/j.jtct.2023.12.174 38826717

[49] Rosenblat TL McDevitt MR Carrasquillo JA Pandit-Taskar N Frattini MG Maslak PG . Treatment of patients with acute myeloid leukemia with the targeted alpha-particle nanogenerator actinium-225-lintuzumab. Clin Cancer Res. (2022) 28:2030–7. doi: 10.1158/1078-0432.ccr-21-3712 35247915 PMC9106874

[50] Finn LE Levy M Orozco JJ Park JH Roboz GJ Tse W . A phase 2 study of actinium-225 (²²⁵Ac)-lintuzumab in older patients with untreated acute myeloid leukemia (AML). *Blood*. (2017) 130(Suppl 1):2638. doi: 10.1182/blood.V130.Suppl_1.2638.2638

[51] Testa U Pelosi E Castelli G . Cd123 as a therapeutic target in the treatment of hematological Malignancies. Cancers Bsl. (2019) 11(9):1358. doi: 10.3390/cancers11091358 31547472 PMC6769702

[52] Castiello MC Bosticardo M Sacchetti N Calzoni E Fontana E Yamazaki Y . Efficacy and safety of anti-cd45-saporin as conditioning agent for rag deficiency. J Allergy Clin Immunol. (2021) 147:309–20:e6. doi: 10.1016/j.jaci.2020.04.033 32387109 PMC8322962

[53] Gustafsson K Rhee C Frodermann V Scadden EW Li D Iwamoto Y . Clearing and replacing tissue-resident myeloid cells with an anti-cd45 antibody-drug conjugate. Blood Adv. (2023) 7:6964–73. doi: 10.1182/bloodadvances.2023010561 37748049 PMC10690556

[54] Srikanthan MA Humbert O Haworth KG Ironside C Rajawat YS Blazar BR . Effective multi-lineage engraftment in a mouse model of fanconi anemia using non-genotoxic antibody-based conditioning. Mol Ther Methods Clin Dev. (2020) 17:455–64. doi: 10.1016/j.omtm.2020.02.001 32226796 PMC7096734

[55] Persaud SP Ritchey JK Kim S Lim S Ruminski PG Cooper ML . Antibody-drug conjugates plus janus kinase inhibitors enable mhc-mismatched allogeneic hematopoietic stem cell transplantation. J Clin Invest. (2021) 131:e145501. doi: 10.1172/jci145501 34730109 PMC8670850

